# Nutritional knowledge, dietary habits and nutritional status of adults living in urban Communities in Lagos State

**DOI:** 10.4314/ahs.v23i1.76

**Published:** 2023-03

**Authors:** FA Olatona, DB Adeniyi, OE Obrutu, AO Ogunyemi

**Affiliations:** 1 Department of Community Health and Primary Care, College of Medicine of the University of Lagos, Lagos, Nigeria; 2 Hubert Department of Global Health, Rollins School of Public Health, Emory University, Atlanta, GA, USA

**Keywords:** Nutritional knowledge, dietary habit, nutritional status of adults

## Abstract

**Background:**

Malnutrition is a major threat to the world's public health. While under-nutrition and micronutrient deficiencies persist, obesity is increasing worldwide. Although malnutrition has been extensively researched among children, it has become of increasing concern among adults because of the relative increase in the prevalence of diet-related non-communicable diseases (NCDs).

**Objectives:**

This study was conducted to determine the nutritional knowledge, dietary habits and nutritional status of adults in an urban community in Lagos State.

**Methods:**

This was a descriptive cross-sectional study involving 320 adults selected using a multi-stage sampling technique. Data was obtained using interviewer-administered questionnaires and standard anthropometric measurements. Chi-square analysis was used to compare prevalence between categories.

**Results:**

Only 15.9% of respondents had good nutritional knowledge. The dietary habits and estimated nutrient intake showed a deficiency of fiber, energy and most micro-nutrients with the exception of zinc, iron and vitamin A. Dietary carbohydrate, protein as well as sodium levels were elevated. BMI findings estimated the prevalence of overweight at 24.8% and obesity at 17.3%. However, there was no statistically significant association observed between nutritional knowledge and status of respondents.

**Conclusion:**

Nutritional knowledge was poor and obesity was relatively high among participants. Improved nutrition education intervention is necessary to increase knowledge and reduce obesity among adults living in urban communities in Lagos, Nigeria.

## Introduction

Nutrition is defined by the World Health Organization (WHO), as the intake of food in relation to the body's dietary needs.^1^ Good nutrition provides a mechanism to promote health and prevent disease across all demographic groups; from infancy through childhood to adulthood, male and female alike.

Malnutrition is a global public health concern and the largest single contributor to disease in the world.^2^ Malnutrition can be in form of undernutrition, micronutrient deficiency or overnutrition/obesity. In Sub-Saharan Africa and Africa as a whole, the prevalence of undernutrition has not significantly changed since 1990 3, whereas the prevalence of global obesity has doubled since 1980;^4^ hence the double burden of malnutrition in this region.

According to the World Food Programme of the United Nations, undernutrition results from inadequate dietary provision of nutrients for growth and maintenance or inadequate utilization of food consumed due to illness.^2^ Problems of malnutrition exist in all countries and cut across socio-economic classes.^5^ Globally, about 795 million people in the world do not have enough food to lead a healthy active life; that is, about one in nine people on earth.^6^ Sub-saharan Africa is the region with the highest prevalence of hunger, with 25% of its population undernourished.^6^ Nutritional knowledge is associated with dietary intake which affects nutritional status.^7^ Prior studies in sub-Saharan Africa have detected that only a quarter of respondents had good nutritional knowledge^8^,^9^. In addition, dietary habits in developing countries are continually being influenced by rapid urbanization and economic growth. These dietary changes include shifts towards more energy dense foods, greater saturated fat intake, reduced intakes of complex carbohydrates, dietary fibre, fruits, and vegetables which translate to poor nutritional status.^10^

While under-nutrition and micronutrient deficiencies persist, the prevalence of overweight, obesity and related NCDs are increasing worldwide.^5^ About 13% of the global adult population are obese while an estimated 3.4 million people die yearly from being overweight and obese.^5^,^11^ According to the WHO, overweight and obesity are linked to more deaths globally than underweight.^11^ Several epidemiological studies have shown a significant association between nutrition and non-communicable diseases (NCDs) such as type 2 diabetes, cardiovascular diseases (CVD), chronic age-related disorders and certain cancers.^12^ Recent estimates suggest that a Body Mass Index (BMI) over 25 kg/m^2^ is responsible for 64% of male and 77% of female cases of type 2 diabetes mellitus worldwide.^9^

Malnutrition and nutrition-based disorders have significant social and economic implications for affected persons and their families. Furthermore, the economic cost of treating NCDs together with the burden they exert on an already frail healthcare system in developing countries cannot be overlooked. In 2002, obesity-related costs in France were estimated at between 2·1and 6·2 billion euros.^13^ In the United States of America, the cost of obesity in 2008 was 147 billion dollars.^14^ Combining obesity-related costs to the already existent undernutrition costs in developing countries would be much worse for their economies.

Although malnutrition has been extensively researched among children (especially under-fives), there is a need for more research in adult populations particularly because of the increasing prevalence of diet-related NCDs in the developing world.

Changing diet into western type has been identified as an important factor that is driving obesity and other diet-related NCDs among adults. These diseases affect quality of life and entail a significant cost to health systems, the economy and households. 13,14,15. Some recent studies in Lagos State determined dietary habits, micronutrient malnutrition and obesity but it was among the university undergraduates and did not explore the association between nutritional knowledge, dietary habits and nutritional status of adult.^16^,^17^ This study seeks to determine nutritional knowledge, dietary habits and nutritional status of adults living in urban area of Lagos State, Nigeria. Findings from the study will be useful for public health practitioners in designing appropriate nutrition interventions for adults living in an urban communities thus reducing morbidity cost and mortality linked with diet-related diseases.

## Methods

A descriptive cross-sectional study was conducted in Apapa, one of the 20 LGAs in Lagos State. Most residents in this area are small and medium scale business owners, civil servants and industrial workers.^18^ A minimum sample size of 258 was calculated using Cochran's formula for single population proportion (n = Z^2^*p*q/ d^2^) using a prevalence of 21.4% of good nutritional knowledge from a previous study^19^,^20^, but 302 respondents were recruited into the study.

A multistage sampling technique was used to select the sample – simple random sampling was used to select Apapa LGA out of the 20 LGAs in Lagos, 4 out of the 12 wards in the LGA, and 2 streets in each ward. All eligible houses on each street were included in the study. Where there was more than one household in a house, one household was selected by simple random sampling. In each selected household, one eligible adult was selected for the study by simple random sampling (balloting). Eligibility was limited to healthy adults between the ages of 18 to 60 years.

A pre-tested interviewer-administered questionnaire was used to obtain data from respondents. The questionnaire was divided into five sections namely: Socio-economic and demographic data, nutritional knowledge, dietary habits, nutrient intake using 24-hour dietary recall and anthropometric measurements.

The knowledge section of the questionnaire was adapted from a standard nutrition knowledge questionnaire and a previous study.^21^,^22^ Nutrient intake was measured using 24-hour diet recall in conjunction with food models and household measures (spoons, cups, bowls and tins) to determine the quantities consumed. Twenty-four-hour diet recall was obtained for two days of the week) one week day and one weekend day) and the mean nutrient intake was calculated. Dietary habits were assessed using a modified food frequency questionnaire.^19^ Anthropometric measurements including weight, height, waist and hip circumference were obtained following standard procedures. Measures were compared to international standards to determine nutritional status.

Four research assistants who are fluent in both English and Yoruba (the prevalent languages in Lagos) were trained successfully on the study design, instruments and data collection method. They conducted the interview along with the researchers. The questionnaire was pretested among 20 adults in a different LGA far away from Apapa (Kosofe).

Data was analysed using Statistical Package for Social Sciences (SPSS Version 17) and Epi-info statistical software. Questions on nutritional knowledge were scored, with 12 being the highest obtainable score. Scores were classified as poor knowledge (0 – 4), fair knowledge (5 – 8), and good knowledge (9 – 12). The 24 hour-diet recall data were analysed using Total Diet Assessment (TDA) software to calculate nutrient intake. Descriptive analysis, such as frequencies, charts, and percentages were used to analyse socio-demographic data and dietary habits. Inferential statistics (chi-square and Fischer's exact) were used to determine the association and differences between mean values.

Ethical approval was obtained from the Health Research and Ethics Committee (HREC) of Lagos University Teaching Hospital, Idi -Araba, Lagos. (The approval no is ADM/DCST/HREC/APP/1098). Official permission was obtained from the Office of the Chairman, Apapa LGA secretariat through the Medical Officer of Health. Informed written consent was obtained from each participant and confidentiality was maintained throughout the study.

## Results

The mean age was 30.40± 9.69 years. Participants were evenly split by gender, religion and marital status, with 49% males, 48% Christians, and 49.3% being married. Majority of respondents were of the Yoruba tribe (73.2%), lived in one-room apartments (83.8%) and about half reported secondary education as their highest level of education (55.0%). Many (39.4%) of the respondents earned a monthly income in the range of N10,001-N20,000 (USD 26.3–52.5), The mean monthly income was N21, 457 (USD 52.3) {SD= N17,888 (USD 43.6)}, 64.9% used bore-hole/Pipe-borne water as their drinking water source, and 68.2% used pit latrine for sewage disposal. (Table 1)

**Table 1:** Socio-demographic characteristics of adults in urban Lagos

Variable	Frequency (n=302)	Percentage (%)
**Age group (years)**		
18 – 30	184	60.9
31 – 40	68	22.5
41–50	36	11.9
51 – 60	14	4.6
Age (years)*	30.4	9.7
**Sex**		
Male	148	49.0
Female	154	51.0
**Religion**		
Islam	151	50.0
Christianity	145	48.0
Traditional	4	1.3
Others	2	0.7
**Marital Status**		
Single	141	46.7
Married	149	49.3
Divorced	3	1.0
Widowed	9	3.0
**Ethnicity**		
Yoruba	221	73.1
Igbo Hausa	48 15	15.9 5.0
Others	18	6.0
**Type of Residence**		
Room apartment	253	83.8
Flat	42	13.9
Bungalow	7	2.3
**Highest level of education**		
None	6	2.0
Primary	66	21.8
Secondary	166	55.0
Tertiary	61	20.2
Vocational	3	1.0
**Number of children**		
0 – 1	171	56.6
2 – 4	120	39.7
5 – 7	11	3.7
**Occupation**		
None	34	11.3
Petty trader	86	28.5
Artisan	118	39.0
Civil servant	51	16.9
Highly skilled Professional	1	0.3
Others	12	4.0
**Water Source**		
Bore-hole/Pipe-borne	196	64.9
Well	105	34.8
Stream	1	0.3
**Sewage Disposal**		
Flush-type WC	95	31.5
Pit latrine	206	68.2
Bush	1	0.3
**Estimated monthly income**		
≤ 10,000	80	26.5
N10,001 – N20,000	119	39.4
N20,001 – N50,000	87	28.8
N50,001 – N100,000	15	5.0
Above N100,000	1	0.3

* Mean (SD)

### Nutritional Knowledge

The majority of the respondents had no knowledge of the average recommended dietary allowance for energy (89.4%) but had correct knowledge about healthy snacking (78.8%). Only 15.9% had overall good nutritional knowledge. (Table 2)

**Table 2:** Nutritional knowledge of adults in urban Lagos

Nutritional knowledge	Respondents with correct responses	
	Frequency	Percentage
Definition of balanced diet	191	63.2
Recommended servings of fruits and vegetables	52	17.2
Food groups that are high in energy	224	74.2
Food groups that are high in protein	229	75.8
Foods that are high in fibre	169	56.0
Foods that are high in calcium	163	54.0
Healthy dietary habit	238	78.8
Recommended Dietary Allowance(energy)	32	10.6
Diet and disease, I	136	45.0
Diet and disease II	143	47.4
Diet and disease III	79	26.2
Diet and disease IV	63	20.9

### Dietary habits

**Figure 1:**
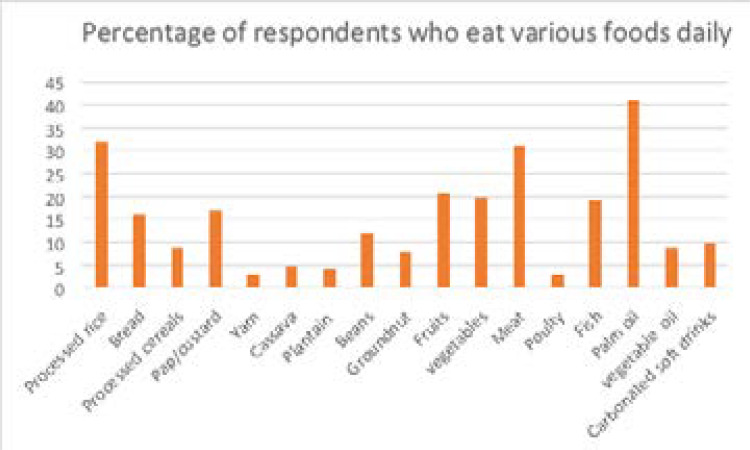
Respondents' Food Consumption Pattern

The majority of the respondents (75.8%) had 3 main meals daily, over half (57.6%) ate breakfast every day. One-third of respondents (33.8%) ate at fast-food restaurants 3-4 times/week and the majority (59.9%) took more than 8 cups of water daily. Rice was the commonest cereal while cassava was the commonest root eaten. (Table 3, Figure 1).

**Table 3:** Dietary habits of adults in urban Lagos

Variable	Frequency (n=302)	Percentage (%)
Number of main meals daily		
Two (2)	49	16.2
Three (3)	229	75.8
Four (4)	18	6.0
Five (5)	6	2.0
**Breakfast eaten**		
Never	5	1.7
1–2 times/week	14	4.6
3–4times/week	42	13.9
5–6times/week	67	22.2
Everyday	174	57.6
**Eating in fast food restaurants/local** **restaurants**		
Never	48	15.9
1–2 times/week	74	24.5
3–4times/week	102	33.8
5–6times/week	55	18.2
Everyday	23	7.6
**Amount of water taken daily in mls (1** **cup=240mls)**		
1–4 cups (240–960ml)	22	7.3
4–8 cups (960–1920ml)	99	32.8
9–12 cups (2160–2880ml)	120	39.7
≥13 cups (≥ 3120ml)	61	20.2
**Total**	**302**	**100**

Generally, diets were deficient in calories (2115±659 for Males, 1867 ±587.0 for females), fibre and all micronutrients except Vitamin A, B6, Sodium, Iron and Zinc. Protein, Carbohydrates, Vitamin A and Sodium were particularly taken in excess by all respondents. (Table 4).

**Table 4:** Respondents' mean energy and nutrient intake by sex and age group

		Male			Female		
Nutrient	Age in years	Mean Intake	RDA	Intake as %RDA	Mean intake	RDA	Intake as %RDA
Energy (Kcal)	18–30	2115±659	2700	78%	1867 ±587.0	2200	85%
	31–50	1937±562	2600	75%	1761 ± 565.7	2000	88%
Protein(g)	18–30	66.4 ±26.4	56	118%	60.6 ±22.5	46	130%
	31–50	62. ±26.2	56	111%	58.7 ± 20.7	46	128%
Carbohydrate(g)	18–30	354 ± 109.2	130	272%	303.2 ±103	130	233%
	31–50	319.1 ± 98.2	130	245%	292.7 ± 104.5	130	225%
Fibre(g)	18–30	7.9 ± 6.3	38	21%	6.6 ± 4.79	25	26%
	31–50	8.3 ± 7.8	38	22%	4.37 ± 3.59	25	17%
Fat(g)	18–30	45.0 ± 20.1	71	63%	40.6± 21.7	56	73%
	31–50	42.91 ± 18.0	69	62%	35.3± 17.5	57	62%
Calcium(mg)	18–30	313 ± 200.09	1000	31%	272.71 ± 238.11	1000	27%
	31–50	270.40 ± 144.00	1000	27%	200.52 ± 123.88	1000	20%
Sodium(mg)	18–30	3495.83 ± 3405	1500	233%	4098 ± 4084.02	1500	273%
	31–50	3406.2± 3353.37	1500	227%	5310.18 ± 4309.89	1500	354%
Potassium(mg)	18–30	1028.25± 716.59	4700	22%	905.91 ± 544.76	4700	19%
	31–50	950.00 ± 782.39	4700	20%	731.89 ± 414.68	4700	16%
Zinc(mg)	18–30	12.97 ± 10.55	7.0	185%	10.78 ± 6.04	4.9	220%
	31–50	16.38 ± 15.03	7.0	234%	12.35 ± 8.67	4.9	252%
Iron(mg)	18–30	25.67 ± 25.62	14	183%	19.93 ± 13.66	29	69%
	31–50	16.77 ± 5.62	14	120%	23.21 ± 22.23	29	80%
Magnesium(mg)	18–30	229.96 ± 104.01	260	88%	236.08 ± 115.65	220	107%
	31–50	226.46±105.6	260	87%	238.03 ± 116.77	220	108%
Vit A (ugRE/day)	18–30	8799.7 ± 7862.98	600	1467%	8172.04±6152.04	500	1634%
	31–60	11,946 ±11,518	600	1991%	10,619.09 ±9,596	500	2123%
Vit C (mg)	18–30	49.10± 49	45	109%	28.65 ±28.18	45	64%
	31–60	48.02±47.08	45	107%	15.01 ±14.82	45	33%
Thiamine (mg)	18–30	1.2 ±0.71	1.2	100%	1.01 ±0.52	1.1	92%
	31–60	1.2 ±0.84	1.2	100%	0.78 ±0.58	1.1	71%
Riboflavin (mg)	18–30	0.90 ±0.54	1.3	69%	0.75 ±0.39	1.1	68%
	31–60	0.9 ±0.62	1.3	69%	0.56 ±0.44	1.1	51%
Pyridoxine (mg)	18–30	0.74 ±0.69	1.3	57%	0.67 ±0.38	1.3	52%
	31–60	0.64 ±0.35	1.3	49%	0.57 ±0.35	1.3	44%
Folate (mg)	18–30	317.39 ±176.28	400	79%	289.66 ±144.52	400	72%
	31–60	297.96 ±204.24	400	74%	233.29 ±142.86	400	58%
Vit B12 (ug)	18–60	1.3 ±0.85	2.4	54%	1.33 ±0.87	2.4	55%
	31–60	1.75 ±1.47	2.4	73%	1.05 ±0.72	2.4	44%

**Figure 2:**
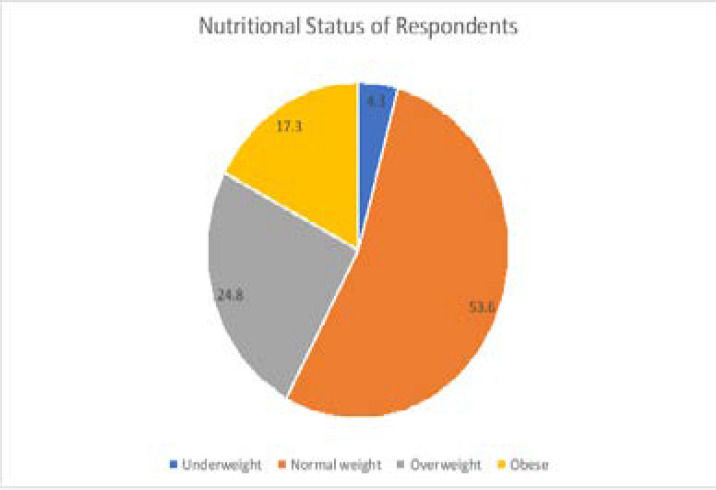
Nutritional Status of adults in urban Lagos

The prevalence of obesity was 17.3%; 53.6% had a normal body mass index, 24.8% were overweight and 4.3% were underweight. Females had a higher prevalence of obesity (20.1%) compared to males (14.2%). (Figure 2)

There were statistically significant associations between respondent's nutritional status and marital status (p=0.008), occupation (p=0.0001), sewage disposal method (p=0.03), and income levels (p=0.001). Most of the respondents with monthly income levels between N50,000 to N100,000 (131USD to 263USD) were overweight/obese. The prevalence of underweight decreased with increasing income while the prevalence of overweight increased with increasing income. There was no statistically significant association between respondents' nutritional knowledge and nutritional status. (Table 5).

**Table 5:** Association between socioeconomic factors and nutritional status of adults in urban Lagos

	Frequency (%)			
Variable	BMI Under-weight (n=13)	Normal (n=162)	Overweight/Obesity (n=127)	p-value
**Marital Status**				
Single	11(7.6)	87(60.0)	47(32.4)	0.008*
Married	2(1.4)	68(46.9)	75(51.7)	
Divorced	0(0)	2(66.7)	1(33.3)	
Widowed	0(0)	5(55.6)	4(44.4)	
**Occupation** a				
None	5(14.7)	23(67.7)	6(17.6)	0.027*
Petty trader	3(3.5)	48(55.8)	35(40.7)	
Artisan	2(1.7)	61(51.7)	55(46.6)	
Civil servant	3(5.9)	25(49.0)	23(45.1)	
Professional	0(0)	0(0)	1(100.0)	
Others	0(0)	5(41.7)	7(58.3)	
**Sewage Disposal** a				
Flush-type (WC)	4(4.2)	34(36.2)	56(59.6)	<0.0001*
Pit latrine	9(4.3)	127(61.7)	70(34.0)	
Bush	0(0)	1(100.0)	0(0)	
**Estimated** **monthly income**				
≤ N10 000	6(7.5)	54(67.5)	20(25.0)	0.001*
N10,000.1–20,000	5(4.2)	64(53.8)	50(42.0)	
20,000.1 – N50,000	2(2.3)	40(46.0)	45(51.7)	
50,000.1 – 100,000	0(0)	3(20.0)	12(80%)	
Above N100,000	0(0)	1(100.0)	0(0)	
**Knowledge**				
Good	3(6.3)	29(60.4)	16(33.3)	0.584
Fair	7(4.6)	77(50.7)	68(44.7)	
Poor	3(2.9)	56(54.9)	43(42.2)	

* Statistically significant (p-value <0.05)

a Fisher's exact used in place of Chi-square

## Discussion

The mean age of respondents in this study was 30.40 ± 9.69 years. This distribution is in keeping with Nigeria having a predominantly young population. 18 The distribution of males (49.0%) and females (51.0%) follows the pattern of the population distribution by sex of Apapa Local Government Area.^18^ The highest level of education of most respondents was secondary level (55%) and the majority (39.1%) were artisans. Both findings are similar to those in a study among adults in South-eastern Nigeria.^23^

Knowledge about diet and related chronic diseases was relatively poor across most respondents. On the contrary, most respondents had knowledge of various food classifications and healthy dietary habits. More than 70% knew energy-giving, bodybuilding and healthy snack foods but only 15.9% had good nutritional knowledge; similar to findings among Iranian adults where 27.4% had good knowledge^24^, and adult mothers in South-west Nigeria where only 19.5% had good knowledge.^9^ Conversely, Taiwan adults had overall nutrition knowledge as high as 62.7%.^25^ Although nutritional knowledge has been linked to dietary habits and nutritional status in other populations,^26^–^28^ we found no statistically significant association between the nutritional knowledge and status of the respondents in this study. While knowledge of proper nutrition is usually the first step in changing dietary practices, our findings imply that knowledge may not directly translate to practice.

Dietary habits showed that among cereals, rice was the most frequently consumed food group. Almost half of respondents reported rice consumption 4 to 6 times a week, while 16.6% ate rice more than once daily. This is similar to findings among Malaysian adults but differs from adult Lebanese who consumed bread most frequently.^29^,^30^ Among the roots and tubers, yam and plantain were most frequently consumed. This is at variance with findings among South-eastern Nigerian adults where cassava was most commonly consumed.^18^ This difference is likely due to the cultural differences among natives of South-Western and South-Eastern Nigeria.

Most respondents consumed fruits and vegetables 1 to 3 times a week. Both leafy and non-leafy vegetables were popularly consumed (59.9% and 50.7% respectively), while 42.1% ate fruits. Among those who had fruits, only 11.9% had fruits every day. A meagre 3.6% ate leafy vegetables daily while 14.9% consumed non-leafy vegetables daily. This is similar to findings from another study among adults in Lagos and Abia state where 27% and 15.6% of respondents consumed fruits and vegetables daily.^23^,^31^ Considering that fruits, and vegetables (in addition to whole grains) are recommended sources of dietary fibre, this pattern reveals a relatively low fibre dietary habits in the study population. At least 5 portions of fruits and vegetables daily are recommended to reduce the risk of deaths from chronic diseases such as heart disease, stroke and cancer.^30^ The inadequate intake of fruits may be explained by the cost of purchasing adequate amounts relative to the income of the study population.

Among meat and milk products, red meat was most commonly consumed similar to findings by Nasreddine et al in Lebanon.^29^ This is contrary to findings from the study in South-east Nigeria and Malaysia where fish was more commonly consumed.^22^,^30^ Red meat is a relatively common and affordable source of protein and saturated fat in this population.^32^ Red palm oil was a commonly used source of fat and oil, and consumed daily by 13.2%.This is akin to the study in South-east Nigeria but different from Nasreddine et al's findings.^23^,^29^ Confectionaries were sparsely taken, pastries moderately consumed (mostly one to three times weekly) while carbonated drinks were commonly consumed four to six times weekly. The major disadvantage of consuming carbonated soft drinks is the excessive consumption of refined sugar which can lead to obesity and other risk factors for NCDs.

A third of respondents ate at fast-food restaurants three to four times a week while one-quarter patronized fast-food restaurants five or more times a week. This correlates with findings from a study among African-American adults. Eating at fast-food restaurants has been associated with higher fat and lower vegetable intake. 34,35 Majority of the respondents (59.9%) drank more than 8 cups of water daily, 75.8% had 3 main meals daily and over half (57.6%) ate breakfast every day. These positive dietary findings are in keeping with findings among adults in a Malaysian nutrition survey.^30^

Estimated nutrient intake among study participants showed deficient intake of fiber, energy and most micronutrients with the exception of zinc, sodium and iron (in males). Vitamin A and pyridoxine were consumed in excess amounts, Vitamin C and thiamine consumption was essentially normal, while consumption of other vitamins was deficient. The low dietary fibre consumption is in keeping with reports from many other studies. 17,31,36 The lower level of iron intake among females is documented as being linked to menstruation and consistent with a British study among adults.^37^

Micro-nutrient deficiency has become increasingly common even in populations already faced with the challenge of undernutrition and overweight/obesity such as Sub-Saharan Africa. The study among urban South African adults also showed that their diet was depleted in minerals and vitamins.^36^ The considerably elevated levels of estimated Vitamin A intake may be related to the frequent use of palm oil in this population. Consumption of sodium was higher than the recommended values for almost all the age groups which may be explained by excessive use of salt and sodium mono-glutamate-containing seasonings which is often culturally acceptable in this population.

Elevated animal protein consumption is associated with kidney disease.^38^ Moreover, since it is usually associated with increased intake of saturated fat, high consumption of red meat increases cardio-metabolic risk factors.^39^ High levels of carbohydrate consumption in this population may be explained by the predominantly starchy foods consumed in the region.

While more than half (53.6%) of respondents had normal weights based on BMI, the mean BMI within the overweight range. The BMI findings estimate a 17.3% prevalence of obesity and 24.8% prevalence of overweight across both sexes. Our observation is similar to findings documented among urban adults in Lagos (21.7%) but much higher than figures obtained in Ile-Ife, Ilorin and Maiduguri (12.5%, 9.8% and 8.1% respectively).^40^–^43^ The prevalence of overweight and obesity in our study is also higher than findings among suburban adults in Jos.^20^ Considering the high carbohydrate and protein consumption as well as fast food patronage in our study population, dietary habits the relatively high prevalence of obesity and overweight is not surprising.

The prevalence of obesity among females was higher than in males; similar to the findings among adults in Ile-Ife. The mean WHR falls just within the normal limit for both sexes. The female sex has been described as a possible predisposing factor for obesity. 41, 43 Waist circumference and weight-hip ratio (WHR) are used to assess abdominal obesity (adiposity), a risk factor for cardiovascular diseases.

There was no statistically significant association between the nutritional knowledge and status of the respondents in this study. This could be explained by the possibility of other constraining factors preventing the translation of knowledge into practice e.g., income level, accessibility and affordability of healthy food options and in some cases, opposing cultural traditions. It may be helpful for further studies to explore the reasons for this discrepancy in order to facilitate the development of interventions to bridge the gap. The statistically significant association between the marital status of the respondents and their nutritional status may be explained by the likelihood of married women developing excess weight during pregnancy and after childbirth. The period of lactation, raising of the infant coupled with a poor exercise culture especially in urban and suburban populations may also play a role. Likewise, married men tend to develop a more sedentary lifestyle compared to their single counterparts. The increasing rate of obesity with higher income observed may be explained by a tendency to spend and consume more calories through processed foods which are more affordable for the affluent. Frequent consumption of fast foods is documented as a contributor to weight gain which leads to excess fat, and this is increasingly popular among adults.^34^

## Conclusion

Nutritional knowledge was poor among adults in the study population. Dietary habits tended to be carbohydrate and protein-rich, high in sodium, but low in dietary fibre and most micro-nutrients. There was a relatively high prevalence of obesity and overweight especially among females. Public health interventions in this population should target increased nutritional education, with explorations of the pathways for better knowledge to translate to dietary practices that improve nutritional status among adults residing in Lagos. Public health policies that involve tangible steps of behavioural interventions to change lifestyle should be formed and implemented.

## Study limitations, strengths and recommendations for further investigation

The cross-sectional study design used for this study would only allow a measure of association. A longitudinal study design would have been preferable to infer causality.

## References

[1] World Health Organization Nutrition [Internet].

[2] United Nations World Food Programme What is malnutrition? | WFP | United Nations World Food Programme - Fighting Hunger Worldwide [Internet].

[3] UNICEF (2007). 2008 The State of the World's Children 2008: Child Survival.

[4] WHO (2011). Obesity and Overweight (Fact Sheet No. 311).

[5] Food and Agriculture Organization of the United Nation Nutrition [Internet].

[6] United Nations World Food Programme Hunger Statistics | WFP | - Fighting Hunger Worldwide [Internet].

[7] Spronk I, Kullen C, Burdon C, O'Connor H (2014). Relationship between nutrition knowledge and dietary intake. Br J Nutr.

[8] Nti CA, Pecku E, Opare-Obisaw C (2015). Nutrition Knowledge, Meal Patterns and Nutritional Status of Energy Drink Users in a Ghanaian University. Journal of Human Ecology.

[9] Akinyinka M, Olatona F, Oluwole E (2016). Breastfeeding Knowledge and Practices among Mothers of Children under 2 Years of Age Living in a Military Barrack in Southwest Nigeria. Int J MCH AIDS IJMA.

[10] WHO/FAO Diet, nutrition and the prevention of chronic diseases: report of a joint WHO/FAO expert consultation, Geneva, 28 January -- 1 February 2002.

[11] World Health Organization Obesity and overweight [Internet].

[12] World Health Organization Diet, nutrition and the prevention of chronic diseases [Internet].

[13] Castetbon K, Vernay M, Malon A, Salanave B, Deschamps V, Roudier C (2009). Dietary intake, physical activity and nutritional status in adults: the French nutrition and health survey (ENNS, 2006 -2007). Br J Nutr.

[14] Finkelstein EA, Trogdon JG, Cohen JW, Dietz W (2009). Annual medical spending attributable to obesity: payer-and service-specific estimates. Health Aff (Millwood).

[15] Abegunde DO, Mathers CD, Adam T, Ortegon M, Strong K (2007). The burden and costs of chronic diseases in low-income and middle-income countries. Lancet.

[16] Olatona FA, Aderibigbe SA, Amu EO, Onabanjo OO, Nnoaham KE (2020). Micro-nutrient related malnutrition and obesity in a university undergraduate population and implications for non-communicable diseases. J Glob Health Rep.

[17] Olatona FA, Onabanjo OO, Ugbaja RN, Nnoaham KE, Adelekan DA (2018). Dietary habits and metabolic risk factors for non-communicable diseases in a university undergraduate population. J Health Popul Nutr.

[18] Population Distribution by Sex, State, LGAs and Senatorial District: 2006 Census Priority Tables (Vol 3) [Internet].

[19] Kadam P, Bhalerao S (2010). Sample size calculation. Int J Ayurveda Res.

[20] Puepet FH, Zoakah AI, Chuhwak EK (2002). Prevalence of Overweight and Obesity Among Urban Nigeria Adults in Jos. Highl Med Res J.

[21] Parmenter K, Wardle J (1999). Development of a general nutrition knowledge questionnaire for adults. Eur J Clin Nutr.

[22] Maziya-Dixon B, Akinyele IO, Oguntona EB, Nokoe S, Sanusi RA, Harris E (2006). National food consumption and Nutritional Survey 2001-2003 Summary IITA.

[23] Ukegbu A, OO M, Onyeonoro U, Chukwuonye I, M A, Ogah O (2013). Food consumption pattern of adult population in Abia State, South East Nigeria. J Community Nutr Health.

[24] Mirmiran P, Mohammadi-Nasrabadi F, Omidvar N, Hosseini-Esfahani F, Hamayeli-Mehrabani H, Mehrabi Y (2010). Nutritional Knowledge, Attitude and Practice of Tehranian Adults and Their Relation to Serum Lipid and Lipoproteins: Tehran Lipid and Glucose Study. Ann Nutr Metab.

[25] Lin W, Hang C-M, Yang H-C, Hung M-H (2011). 2005-2008 Nutrition and Health Survey in Taiwan: the nutrition knowledge, attitude and behavior of 19-64-year-old adults. Asia Pac J Clin Nutr.

[26] Jeruszka-Bielak M, Kollajtis-Dolowy A, Santoro A, Ostan R, Berendsen AAM, Jennings A (2018). Are Nutrition-Related Knowledge and Attitudes Reflected in Lifestyle and Health Among Elderly People? A Study Across Five European Countries. Front Physiol [Internet].

[27] Alaunyte I, Perry JL, Aubrey T (2015). Nutritional knowledge and eating habits of professional rugby league players: does knowledge translate into practice?. J Int Soc Sports Nutr.

[28] Argôlo D, Borges J, Cavalcante A, Silva G, Maia S, Moraes A (2018). Poor dietary intake and low nutritional knowledge in adolescent and adult competitive athletes: a warning to table tennis players. Nutr Hosp.

[29] Nasreddine L, Hwalla N, Sibai A, Hamzé M, Parent-Massin D (2006). Food consumption patterns in an adult urban population in Beirut, Lebanon. Public Health Nutr.

[30] Norimah AK, Safiah M, Jamal K, Haslinda S, Zuhaida H, Rohida S (2008). Food Consumption Patterns: Findings from the Malaysian Adult Nutrition Survey (MANS). Malays J Nutr.

[31] Olatona F, Sosanya A, Sholeye O, Obrutu O, Nnoaham K, Olatona F A, Sosanya A, Sholeye OO, Obrutu OE, Nnoaham KE (2018). Knowledge of fruits and vegetables, consumption pattern and associated factors among adults in Lagos State, Nigeria. Res J Health Sci.

[32] The Nutrition Source (2012). Vegetables and Fruits [Internet].

[33] Ogunwole OA, Adedeji BS (2014). Consumers' Preference and Perception of the different Types of Meat among Staff and Students of the University of Ibadan, Nigeria. J Agric Environ Sci.

[34] Bowman SA, Vinyard BT (2004). Fast Food Consumption of U.S. Adults: Impact on Energy and Nutrient Intakes and Overweight Status. J Am Coll Nutr.

[35] Satia JA, Galanko JA, Siega-Riz AM (2004). Eating at fast-food restaurants is associated with dietary intake, demographic, psychosocial and behavioural factors among African Americans in North Carolina. Public Health Nutr.

[36] Bourne LT, Langenhoven ML, Steyn K, Jooste PL, Laubscher JA, Van der Vyver E (1993). Nutrient intake in the urban African population of the Cape Peninsula, South Africa. The Brisk study. Cent Afr J Med.

[37] Friedman AN, Yu Z, Juliar BE, Nguyen JT, Strother M, Quinney SK, Li L, Inman M, Gomez G, Shihabi Z, Moe S (2010). Independent influence of dietary protein on markers of kidney function and disease in obesity. Kidney Int.

[38] Zhubi-Bakija F, Bajraktari G, Bytyçi I, Mikhailidis DP, Henein MY, Latkovskis G, Rexhaj Z, Zhubi E, Banach M, International Lipid Expert Panel (ILEP) (2021). The impact of type of dietary protein, animal versus vegetable, in modifying cardiometabolic risk factors: A position paper from the International Lipid Expert Panel (ILEP). Clin Nutr.

[39] Vyas A, Greenhalgh A, Cade J, Sanghera B, Riste L, Sharma S (2003). Nutrient intakes of an adult Pakistani, European and African-Caribbean community in inner city Britain. J Hum Nutr Diet.

[40] Amira C, Sokunbi D, Dolapo D, Sokunbi A, Amira CO, Sokunbi DOB, Dolapo D, Sokunbi A (2011). Prevalence of obesity, overweight and proteinuria in an urban community in South West Nigeria. Niger Med J.

[41] Adedoyin RA, Mbada CE, Balogun MO, Adebayo RA, Martins T, Ismail S (2009). Obesity prevalence in adult residents of Ile-Ife, Nigeria. Nig Q J Hosp Med.

[42] Desalu O, Salami A, Oluboyo P, Olarinoye J Prevalence and socio-demographic determinants of obesity among adults in an urban Nigerian population. Sahel Med J.

[43] Oyeyemi AL, Adegoke BO, Oyeyemi AY, Deforche B, De Bourdeaudhuij I, Salis J Environmental factors associated with overweight among adults in Nigeria. Int J Behav Nutr Phys Act.

